# Ebola outbreak in rural West Africa: epidemiology, clinical features and outcomes

**DOI:** 10.1111/tmi.12454

**Published:** 2015-02-03

**Authors:** Silvia Dallatomasina, Rosa Crestani, James Sylvester Squire, Hilde Declerk, Grazia Marta Caleo, Anja Wolz, Kathryn Stinson, Gabriela Patten, Raphael Brechard, Osman Bamba-Moi Gbabai, Armand Spreicher, Michel Van Herp, Rony Zachariah

**Affiliations:** 1 Médecins Sans FrontièresFreetown, Sierra Leone; 2 Médecins Sans Frontières, Ebola Task Force, Brussels Operational CentreBrussels, Belgium; 3 Ministry of Health and Sanitation, District Health ServicesKailahun, Sierra Leone; 4 Manson Unit, Médecins Sans FrontièresLondon, UK; 5 School of Public Health & Family Medicine, University Cape TownCape Town, South Africa; 6 Operational Research Unit, Brussels Operational Centre, Médecins Sans FrontièresLuxembourg, Luxembourg

**Keywords:** Ebola, Kailahun, operational research, MSF, health workers

## Abstract

**Objective:**

To describe Ebola cases in the district Ebola management centre of in Kailahun, a remote rural district of Sierra Leone, in terms of geographic origin, patient and hospitalisation characteristics, treatment outcomes and time from symptom onset to admission.

**Methods:**

Data of all Ebola cases from June 23rd to October 5th 2014 were reviewed. Ebola was confirmed by reverse-transcriptase-polymerase-chain-reaction assay.

**Results:**

Of 489 confirmed cases (51% male, median age 28 years), 166 (34%) originated outside Kailahun district. Twenty-eight (6%) were health workers: 2 doctors, 11 nurses, 2 laboratory technicians, 7 community health workers and 6 other cadres. More than 50% of patients had fever, headache, abdominal pain, diarrhoea/vomiting. An unusual feature was cough in 40%. Unexplained bleeding was reported in 5%. Outcomes for the 489 confirmed cases were 227 (47%) discharges, 259 (53%) deaths and 3 transfers. Case fatality in health workers (68%) was higher than other occupations (52%, *P *=* *0.05). The median community infectivity time was 6.5 days for both general population and health workers (*P *=* *0.4).

**Conclusions:**

One in three admitted cases originated outside Kailahun district due to limited national access to Ebola management centres – complicating contact tracing, safe burial and disinfection measures. The comparatively high case fatality among health workers requires attention. The community infectivity time needs to be reduced to prevent continued transmission.

## Introduction

Ebola virus disease (EVD), also called Ebola haemorrhagic fever or simply Ebola, is a disease of humans and other primates caused by viruses of the family filoviridae [1, 2]. It was first discovered in 1976 close to the Ebola River in the Democratic Republic of Congo from which it gets its name. Fruit bats are believed to be the virus reservoir, able to spread the virus without being affected [3]. Humans become infected by contact with bats or a living or dead animal that has been infected by bats. This then leads to human outbreaks [1]. Case fatality can be as high as 90% and despite four decades of documented existence there is no specific licensed treatment for Ebola. It is thus considered a neglected tropical disease [4].

The incubation period lasts 2–21 days and pathogenesis involves immune suppression and a systemic inflammatory response that causes impairment of the vascular, coagulation and immune systems, leading to multiorgan failure [1]. Virus transmission during outbreaks occurs through transmission in the community between household members, close contacts and caregivers of Ebola patients [5, 4], contact with dead bodies or body fluids during funeral ceremonies [6], and in health facilities through breaches in barrier nursing and contamination of medical equipment [7, 8]. The response comprises early diagnosis, isolation and supportive medical care for confirmed cases; contact tracing through daily surveillance of contacts; active case-finding and alert investigations; safe burial practices and home disinfection; and health promotion to ensure community acceptance of these measures. Ensuring that these activities are conducted in a safe manner requires staff to be trained in infection control and equipped with personal protective equipment [7, 8].

On 8 August 2014 WHO declared the West African Ebola epidemic in Guinea [9], Liberia, Nigeria, Senegal and Sierra Leone a ‘public health emergency of international concern’ – this being the largest outbreak in history [10, 11]. Sierra Leone is one of the worst-affected countries with a doubling of cases every 30 days as of September 2014.

Although WHO has analysed the epidemiologic characteristics of the outbreak using multicountry data from West Africa, [11] no analysis has focused on a rural district setting. Knowledge of the epidemiologic characteristics of the outbreak in hard-to-reach rural areas with considerable logistic and resource challenges is vital to identify gaps in control efforts and inform an effective response. Since June 2014, Médecins Sans Frontières has managed the Ebola management centre in the rural district of Kailahun in Sierra Leone. During the first 4 months (June to October 2014) of the outbreak, we assessed trends in admissions of Ebola cases to the Ebola management centre in terms of geographic mapping, patient and hospitalisation characteristics, treatment outcomes and community infectivity time.

## Methods

This observational study in November 2014 included all patients who were enrolled consecutively at arrival at the Ebola management centre between 23rd June and 5th October 2014. Follow-up was censured on 10th November 2014.

### Study setting and site

Sierra Leone has an estimated population of six million and despite decades of mining of diamonds, titanium, bauxite and gold, 70% of its people live in poverty [12]. The 1991–2002 civil war devastated the country and its health system; Sierra Leone ranks 5th highest for maternal mortality and 11th for infant mortality worldwide [12]. Even before the Ebola outbreak, which resulted in the deaths of many health workers, there were only 0.2 doctors and 1.7 nurses per 10 000 population, mostly located in urban areas [12]. The study site was the only Ebola management centre in Kailahun town in rural Kailahun district of Sierra Leone, which has 400 000 inhabitants and a surface area of 4859 km^2^. It lies in the north-east of Sierra Leone and is bordered by Liberia to the east and Guinea to the north.

### Ebola control at district level

The district health management team of the Ministry of Health and Sanitation is responsible for overall coordination of Ebola control activities and partners. Partners include the International Federation of the Red Cross (involved with safe burials and home disinfection), Save the Children (contact tracing), WHO (safe burials, support to contact tracing, surveillance, logistic support and training), the World Food Programme (providing food for households under quarantine), the National Microbiological Laboratory, Winnipeg, Canada (laboratory diagnosis of Ebola) and MSF (management of the Ebola management centre, health promotion). A national Ebola call centre receives alerts and despatches teams to investigate and implement control activities. This includes investigating alerts of suspect cases and deaths in the community. At the time of this report, only three dedicated ambulances were available in the whole district for patient transfers to the Ebola management centre.

### The MSF Ebola Management Centre

Confirmed and suspected Ebola cases from Kailahun and those referred from neighbouring districts were admitted to the Ebola management centre, which progressively increased its bed capacity from 72 to 94 beds. The set-up and functioning of the centre has been previously described [13]. In brief, approximately 25 people per day – doctors, nurses, disinfection teams, cooks, cleaners, health promotion, counselling teams and logisticians – ensure six-hourly shifts. The centre has its independent water supply, 24-h electricity supply and an on-site kitchen.

Patients arrive by ambulances and are assessed in a triage area. Their clinical signs are then recorded, and they are admitted to the suspect or probable area of the centre depending on their case classification [13]. All cases undergo on-site laboratory confirmation by real time polymerase chain reaction (RT-PCR, Public Health Agency, Winnipeg, Canada). Confirmed Ebola cases are then moved to the confirmed area of the centre.

Supportive care is provided until the PCR turns negative. Those with two negative PCR tests for Ebola are discharged to seek care from the general health services. All cases receive a systematic course of antimalarials, a broad-spectrum antibiotic and symptomatic care for fever, diarrhoea and vomiting.

Treatment outcomes were standardised and documented as recovered (showed clinical improvement and was discharged PCR-negative); death after being admitted; abandoned (left without medical consent); transferred (transferred to another facility). A patient admitted as an Ebola suspect but found negative on repeated PCRs was classified as a non-case.

### Data collection and statistical analysis

An epidemiologist gathered data from patient files daily and encoded them into a password-protected database used for the analysis. Information on contacts was sourced from the district health office. Treatment outcomes for the period June 23rd to October 5th 2014 were censured on 10th November 2014.

The cumulative incidence of death was estimated and expressed graphically using the Kaplan–Meier method. The number of days from onset of symptoms to admission at the Ebola management centre was considered the community infectivity time. Differences between groups were compared using chi-square and Wilcoxon Rank-sum test. The level of significance was set at *P *≤* *0.05, and 95% confidence intervals (CIs) were used. Data analysis was performed using STATA 11 software (Stata Corporation, College Station, TX, USA).

### Ethical considerations

The study used data collected during surveillance and response activities for Ebola at district level and stripped of patient identifiers. Informed consent was not applicable. The study satisfied the MSF Ethics Review Board criteria for studies using routinely collected data (Geneva, Switzerland); the Ebola interventions were approved by the Ministry of Health, Sierra Leone.

## Results

### Trend in admissions and geographic origin of cases

**Figure 1:**
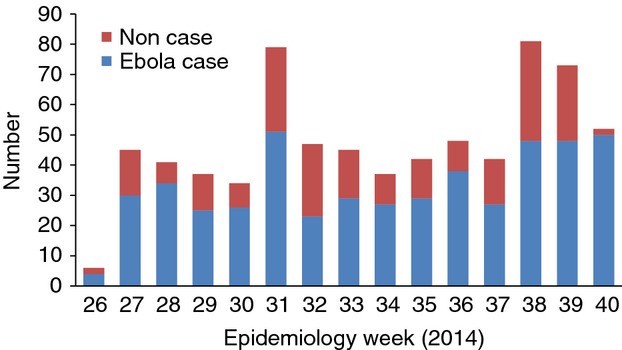
Trend in admissions to the Ebola management centre stratified by confirmed Ebola case and non-case, Kailahun, Sierra Leone during Epidemiology weeks 26–40 (June 23rd – October 5th 2014).

Figure1 shows the trend in admissions stratified by Ebola cases and non-cases. Of 709 patients admitted to the Ebola management centre with clinical features suggestive of Ebola, 220 (31%) were declared non-cases after PCR testing and discharged (median hospitalisation time 2 days, IQR 1–3).

**Figure 2:**
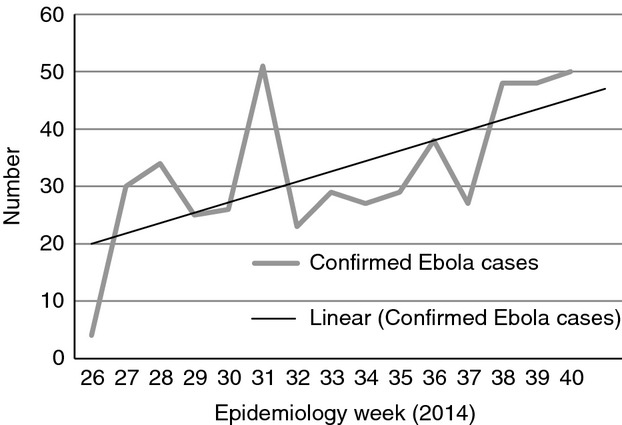
Trend in confirmed Ebola cases admitted to the Ebola management centre in Kailahun, Sierra Leone during Epidemiology weeks 26–40 (June 23rd – October 5th 2014).

**Figure 3:**
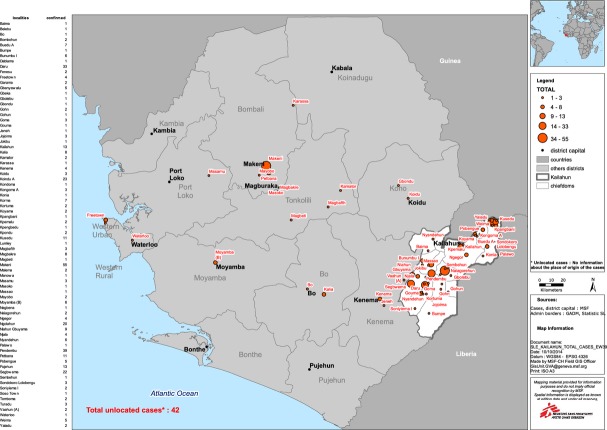
Mapping of confirmed Ebola cases admitted to the Ebola management centre in Kailahun, Sierra Leone during Epidemiology weeks 26–40 (June 23rd – October 5th 2014). Area in white represents Kailahun district, Sierra Leone; Orange circles represent Ebola cases, the size of the circle being proportional to the number of cases.

Figure2 shows the upward and erratic trend in confirmed cases of Ebola admitted to the EMC. This trend is influenced by ambulance availability for referrals and cases from other districts. Figure3 shows the widespread origin of Ebola cases from all over Sierra Leone. Of 489 confirmed cases, 166 (34%) originated from districts other than Kailahun – some as far as Freetown, 416 km and 8–9 h away.

### Patient characteristics

Of 489 confirmed cases, 250 (51%) were male. The median age was 28 years (IQR, interquartile range: 17–40 years, range 3 months – 80 years). Only 3 individuals had eaten bush meat, while 76 (16%) had attended a funeral in the previous 21 days. The great majority (401) of cases were thus likely related to direct contact with body fluids of infected individuals at home or in the community.

Twenty-eight health workers were admitted with Ebola (19 from Kailahun district and 12 from other districts) comprising 11 nurses, 7 community health workers, 2 doctors, 2 laboratory technicians, an ambulance driver, a vaccinator, a national staff working for an international organization a contact tracer, a counsellor and a surveillance officer.

### Clinical manifestations

**Figure 4:**
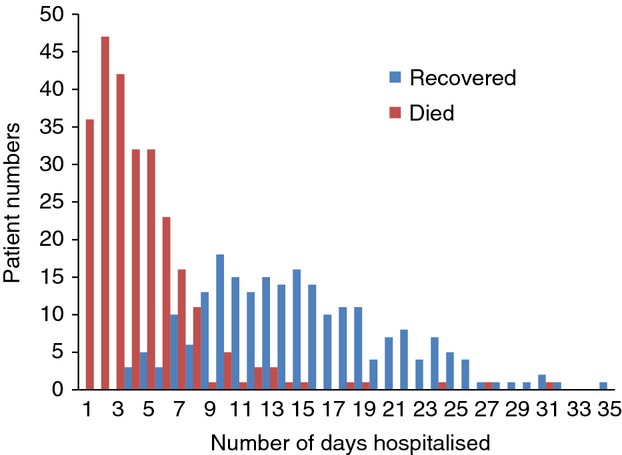
Length of hospitalisation of confirmed Ebola cases among those who died or recovered at the Ebola management centre, Kailahun, Sierra Leone during Epidemiology weeks 26–40 (June 23rd – October 5th 2014).

Table1 shows the clinical features of 245 consecutive Ebola patients for whom clinical history was recorded consecutively from August 2014. Fever, fatigue, headache, joint and abdominal pains, diarrhoea and vomiting were present in >50%. Unusual features were chest pain in 44% and cough in 40%. Unexplained bleeding was seen in only 5%. The median hospitalisation time (Figure4) for all 489 confirmed cases was 7 days (IQR 3–14, range 1–35). For patients who died this was 4 days (IQR 2–6, range 1–31), for patients who recovered, 14 days (IQR 11–19, range 4–35).

**Table 1:** Clinical features at admission of confirmed Ebola cases recorded consecutively at the Ebola management centre, Kailahun, Sierra Leone (August–October 2014)*

Clinical features (*n*-245)	(%)
Fever	87
Fatigue	77
Headache	73
Anorexia	72
Joint pain	56
Abdominal pain	51
Diarrhoea	48
Nausea/vomiting	46
Chest pain	44
Cough	40
Difficulty swallowing	26
Sore throat	26
Difficulty breathing	20
Hiccups	15
Pain behind the eyes	12
Confused/disoriented	9
Jaundice	8
Unexplained bleeding	5
Skin rash	3
Conjunctivitis	2
Coma	1

* Clinical history was recorded consecutively only from August 2014 when such data collection was initiated in the project.

### Case fatality and treatment outcomes

Of the 489 admitted Ebola cases, one outcome was unrecorded in the database. Of the remaining 488, 227 (47%, 95% CI: 42–51) were discharged, 259 died (53%, 95% CI:49–58), and 3 (1%) were transferred.

**Figure 5:**
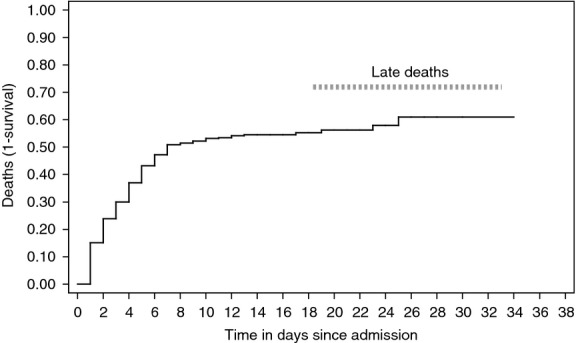
Cumulative incidence of death among confirmed Ebola cases admitted to the Ebola management centre, Kailahun, Sierra Leone during Epidemiology weeks 26 to 40 (June 23rd – October 5th 2014).

Figure5 shows the cumulative incidence of death among confirmed Ebola cases. 246 (95%) deaths occurred within the first 10 days of admission and 8 after 10–18 days. Five patients died after at least 18 days (including one at 31 days), when they were in the recovery or convalescent phase (defined as late deaths). Exact reasons for these late deaths are unknown.

Of the 28 health workers admitted, 19 (68%) died (2 doctors and 2 laboratory technicians, 7 of the 11 nurses, 3 of the 7 community workers, the ambulance driver, the vaccinator, a national staff working for an international organisation, a counsellor and a surveillance officer). Case fatality in health workers (68%, 95% CI: 48–84) was higher than in other occupations (52%, 95%CI: 47–57) (*P = *0.05).

### Contact tracing and community infectivity time

In addition to 489 confirmed Ebola cases, there were 36 probable cases – 19 deaths on ambulance arrival and 17 unexplained community deaths. The number of contacts for all 525 cases was estimated at 5250, of whom 1923 (37%) were identified. The median community infectivity time (CIT) was 6.5 days (IQR 3–8 days) for both general population and health workers (*P *=* *0.4). Median CIT was 4 days (IQR, 3–7) for cases from Kailahun district and 8 days (IQR, 5–11) for those from other districts (*P* < 0.001).

## Discussion

This is the first study from a remote rural district setting in Sierra Leone during the current Ebola outbreak. It shows an upward trend in Ebola admissions with one in three cases originating from outside the district. Although about half of all admitted Ebola cases recovered, the case-fatality rate among health workers was high. Of particular concern is the long community infectivity time, which magnifies community transmission.

Our findings reflect the reality of a district setting with very limited logistics and few trained staff struggling to cope with the scale of the outbreak. It also points to inadequate infection control and protection of health workers within an already weak health system eroded by decades of conflict.

The strengths of the study are that dat came from an operational setting and likely to reflect the ground reality; treatment outcomes were standardised; and on-site epidemiologists collected the data. There was also ready access to Ebola laboratory testing, a key component in managing cases, and MSF has considerable experience in managing Ebola from previous epidemics. However, there is likely to be significant underreporting [10, 14] of cases for various reasons: data on confirmed cases only included those were admitted to the Ebola management centre, but not those who could not be admitted when bed capacity was reached. Dire shortages of dedicated ambulances – three for a district population of almost half a million – meant that many cases never made it to the Ebola management centre. Stringent government-mandated quarantine and resulting household food shortages reportedly led to hiding of possible Ebola cases, stigma, distrust and even hostility towards health workers. This complicated surveillance and identification of new clusters [15]. Development of social acceptance through health promotion and careful collaboration with communities leading to understanding of anthropological perspectives is vital but was missed in the early stages of launching control interventions. Research in this area is urgently required [16].

Despite these limitations, our findings raise a number of important operational observations. First, more than one in three confirmed Ebola cases originated from outside Kailahun district as most districts did not have functioning Ebola management centre. The community infectivity time was significantly higher for those originating from other districts, which fostered transmission. No formal coordination system for surveillance and reporting between districts existed. These shortcomings complicated contact tracing, safe burial procedures and disinfection measures at sites beyond the jurisdiction of a given district. Transportation of recovered patients and corpses to their homes was a challenge. An issue of serious concern to heath workers and the community at large was that burial of corpses from distant locations often occurred without the presence of loved ones. These problems highlight the necessity of having at least one Ebola management centre in every district, something that still has not been achieved.

Second, a third of patients arriving at the Ebola management centre were non-cases, putting them at potential risk of Ebola cross-infection. To avoid this, ambulances should not transport several patients at once, the quality of triage before admission must improve and partitions between patients in the suspect area must be introduced. Have a point-of-care rapid diagnostic test that excludes non-Ebola cases would considerably reduce unnecessary admissions, although probable cases who present early would still need to be admitted for a repeat PCR test after 48–72 h.

Third, the unusual observation that almost half of all confirmed cases presented with a cough [1, 17] poses a risk of cross-infection due to droplet propulsion between patients and clinical staff during triage. Face masks need to be given to patients on arrival and protection measures for staff improved.

Fourth, the majority of deaths occurred within 10 days of admission when supportive therapy is likely to impact survival. Unfortunately, the scale of the epidemic, the very difficult working environment, and the need to ensure maximum safety for health workers limited the use of invasive procedures such as intravenous fluids and/or aggressive correction of electrolyte imbalances when the workload became high. Innovative ways of providing intensive supportive care that do not compromise the safety of health workers are needed.

The issue of late deaths during the recovery phase is particularly disheartening to medical teams. It may also raise suspicion, fear and stigma against the Ebola management centre and hamper acceptability by the community. Cardiac arrhythmias (as a result of severe electrolyte imbalances), myocarditis, pericardial effusions, encephalitis or other reasons may be to blame. Research into the cause of late mortality is important and should be pursued. There might also have been associated comorbidity (e.g. HIV) that we were unaware of.

Finally, of serious concern is that 28 health workers were affected and suffered a higher case fatality than others. Surprisingly, health workers did not present earlier than the general population, which is worrying as symptomatic health workers are at high risk of both acquiring and transmitting infection. In a country that had only 0.2 doctors and 1.7 nurses per 10 000 inhabitants before the epidemic, additional staff losses are devastating and will have long-term repercussions for an already weak, crippled health system [18, 14]. Measures to improve health monitoring of staff are needed. On a wider scale, the number of infected health workers who died in West Africa from Ebola (592 infected and 340 deaths) shows that the medical community seems to have placed inadequate emphasis on safety and advocacy for health workers [14, 19, 20].

Focused immediate and long-term attention on a national scale is needed to ensure infection control and protection for the remaining health workers. Curbing the population spread of Ebola plus new vaccines and treatments would provide a safety net for health workers and need to be fast-tracked [21]. Compensating for Ebola-related health worker attrition through accelerated training of new and adapted health worker cadres is urgently needed but dependent on international support [22, 18].

## Conclusion

Despite severe limitations, commendable efforts have been made towards trying to control the Ebola outbreak in a rural district of Sierra Leone. The recovery rate of almost 50% in difficult circumstances is positive, but balanced by a significant loss of healthcare workers, woeful inadequacies of transport and case-finding and difficult access to the Ebola management centre. Urgent and sustained action by national and international partners is necessary to support Ebola management centres in all districts, solve operational problems and contain the epidemic.
